# Building a learning community of Australian clinical genomics: a social network study of the Australian Genomic Health Alliance

**DOI:** 10.1186/s12916-019-1274-0

**Published:** 2019-02-22

**Authors:** Janet C. Long, Chiara Pomare, Stephanie Best, Tiffany Boughtwood, Kathryn North, Louise A. Ellis, Kate Churruca, Jeffrey Braithwaite

**Affiliations:** 1 Australian Institute of Health Innovation, Macquarie University, Sydney, Australia; 2 Murdoch Children’s Research Institute, Melbourne, Australia; 3 Australian Genomics Health Alliance, Melbourne, Australia

**Keywords:** Social network analysis, Complexity science, Systems change, Genomics, Implementation, Dissemination, Learning community

## Abstract

**Background:**

Adopting clinical genomics represents a major systems-level intervention requiring diverse expertise and collective learning. The Australian Genomic Health Alliance (Australian Genomics) is strategically linking members and partner organisations to lead the integration of genomic medicine into healthcare across Australia. This study aimed to map and analyse interconnections between members—a key feature of complexity—to capture the collaborations among the genomic community, document learning, assess Australian Genomics’ influence and identify key players.

**Methods:**

An online, whole network study collected relational data from members asking them about two time points: baseline, before Australian Genomics started operation in 2016 and current in 2018. Likert style questions assessed the influence of various sources of knowledge on the respondents’ genomic practice. A secure link to the online questionnaire was distributed to all members of Australian Genomics during May 2018. Social network data was analysed and visually constructed using Gephi 0.9.2 software, and Likert questions were analysed using chi-squared computations in SPSS. The project was given ethical approval.

**Results:**

Response rate was 57.81% (222/384). The genomic learning community within Australian Genomics was constructed from the responses of participants. There was a growth in ties from pre-2016 (2925 ties) to 2018 (6381 ties) and an increase in density (0.020 to 0.043) suggesting the strong influence of Australian Genomics in creating this community. Respondents nominated 355 collaborative partners from 24 different countries outside of Australia and 328 partners from within Australia but outside the alliance. Key players were the Australian Genomics Manager, two clinical geneticists and four Operational staff members. Most influential sources of learning were hands on learning, shared decision making, journal articles and conference presentations in contrast to formal courses.

**Conclusions:**

The successful implementation of clinical genomics requires the engagement of multidisciplinary teams across a range of conditions and expertise. Australian Genomics is shown to be facilitating this collaborative process by strategically building a genomic learning community. We demonstrate the importance of social processes in building complex networks as respondents name “hands on learning” and “making group decisions” the most potent influences of their genomic practice. This has implications for genomic implementation, education and work force strategies.

## Background

A useful approach to understanding across-the-board adoption of new health technologies is to view healthcare as a complex adaptive system (CAS) [1, 2]. This term is used to describe a collection of agents (individuals) that interact dynamically and who may, as a result, learn, adapt and reorganise themselves without substantial outside direction. CASs display a set of features that, if better understood, can be leveraged (or mitigated) in any change or improvement activities [3–5]. Hallmark characteristics of CASs are the multiple agents whose actions are not always predictable and whose relationships and interdependencies change the context in which they work [2, 6]. Over time, these connections and influences can give rise to a tipping point, with new emergent behaviours, and self-organisation into new team configurations [7, 8]. In the language of complexity science, this might become a phase transition to a new order [7]. A glossary of complexity and social network terms used in this paper is provided in Table 1.

**Table 1:** Glossary of complexity and social network terms

Complexity terms	
Agents	Individual components that make up a system; people who act independently in social systems. Here they are the individual members of Australian Genomics.
Complex adaptive system (CAS)	Term used for a collection of agents that interact dynamically and whose interactions and interdependencies may lead to learning, adaptation and emergent behaviours.
Phase transition	A time when the system undergoes a crucial change or reaches a tipping point in which a significant transformation in how agents are organised or interact starts. This can alter the system, or the context in which the agents operate.
Self-organisation	The tendency for agents in a CAS to interact in certain ways and form semi-formal groups without undue outside direction.
Social network terms	
Betweenness centrality	A measure of the influence of an actor in connecting others in the network. Actors with high betweenness centrality lie most often on the shortest path between other nodes. Betweenness centrality positions the actor to be a go-between or broker.
Centralisation	A network measure that shows how dominated the whole network is by one or more nodes in terms of their ties. Low centralisation indicates a more even distribution of ties.
Density	The proportion of ties found across a network per the number of possible ties. Expressed as a number between 0 and 1.0, when 1.0 means all possible ties are present (everyone is connected to everyone else).
In-degree	Number of ties directed to a node, i.e. the number of times a particular individual is nominated by others as having that relationship with them. A measure of influence, importance or accessibility.
Nodes	Agents or individuals. Depicted as points or small circles in sociograms
Out-degree	Number of ties a particular node directs to other nodes, i.e. the number of other people a particular individual nominates as having that relationship to them. A measure of connectedness.
Sociogram	A graphical depiction of the relationship data in a social network study collected from individuals and then collated. Based on graph theory, parameters can be computed from the aggregated data.
Ties	The relationship of interest in a social network study. Depicted as a line between nodes. Two nodes are said to be tied if one or both acknowledge the relationship.

Clinical genomics (the sequencing of all, or significant portions of a patient’s genes to aid diagnosis and management of conditions) has increasingly been shown as being cost effective and having clinical utility across many different conditions [9]. Globally, government funding for genomic programs is estimated at US$4 bill involving 14 countries [10]. Genomics has applications across all major clinical specialties from oncology to neonatology, urology to cardiology. Successful adoption of clinical genomics relies on interdisciplinary teams of clinical specialists, laboratory scientists, genetic specialists and counsellors [11]. These emerging teams and the increasingly complex interactions between team members are clear features of complexity.

### Developments in Australia

The Australian Genomic Health Alliance [12] (hereafter, Australian Genomics) is strategically bringing together a wide range of partner individuals, teams, organisations and peak agencies to lead the integration of genomic medicine into healthcare across Australia. Arising from a successful bid (AUD$25 M over 5 years) to the National Health and Medical Council targeted call for *Research into Preparing Australia for the Genomics Revolution in Health Care*, it has four Programs of activity (National Diagnostic and Research Network; Data Sharing and Data Management; Health Economics, Implementation, Policy and Ethics; Education and Workforce). In addition, multiple clinical Flagship Projects have been established. These competitively funded projects aim to build evidence of the intervention of genomic testing (e.g., cost-effectiveness, clinical utility and patient acceptability) within a discrete condition (e.g., cardiovascular), life stage (e.g., neonates), or group of syndromes (e.g., intellectual disability). There have been 17 Flagship projects established to date.

The Australian States and Territories have differing approaches to government funding, and whether or not to fund, clinical genomic testing. Some have invested in local genomic research projects. State-based Genomic Alliances are also therefore part of the Australian genomic context. The National and State-based alliances, while similar in their aims, and overlapping in membership, collaborate rather than compete via multiple governance mechanisms including a national steering committee.

As a nationwide alliance of clinicians, scientists, researchers and consumers across varied sites, conditions and contexts, Australian Genomics may be considered a CAS in itself and be expected to display CAS features such as interdependencies, emergent behaviours, sensemaking, learning and adaptation among the agents [6, 8, 13, 14]. This current project is part of a larger study looking at Australian Genomics as a CAS. Understanding social processes is an important part of CASs, so this study sought to demonstrate how learning communities were forming, facilitated by Australian Genomics. Understanding these processes and leveraging their inherent social structure is crucial to their role as facilitators of progress, sharing of expertise and dissemination of ideas.

Learning is a highly social process [15, 16], especially in clinical genomics, where interdisciplinary teams must work together to be successful. To learn about genomics, build the necessary knowledge and skills based across siloed specialties, and deliver safe, high-quality genomic medicine, a reconfiguration of teams is needed to pool expertise. In other words, there is a need to combine medical physicians with expertise on clinical presentations and medical management, genetic specialists with expertise in the manifestation and course of genetic conditions, laboratory and medical scientists with expertise in analysis and variant curation, and implementation scientists with skills in change and improvement. Given enough time, a key feature of a CAS may manifest: self-organisation, as genomic users in different specialties seek out one another. In such circumstances, individual agents learn from one another and become part of a learning community or knowledge exchange network. Indeed, it was an early observation by senior members of Australian Genomics that people from formerly siloed disciplines were now collaborating and learning from one another. Australian Genomics is leveraging this natural tendency inherent in many socio-professional groupings to learn from others and collaborate synergistically, accelerating it by bringing genomic practitioners together around a strategic goal: “to provide evidence for the equitable, effective and sustainable delivery of genomic medicine in healthcare” [12].

### Social network studies

At the heart of complexity science approaches, social network research is a way to explore and build a picture of relationships such as collaborative clusters or communication pathways within a specified group of people or sites [17, 18]. It can be used diagnostically to address gaps, silos and informational bottlenecks; to map changes in relationships over time; and to identify key people in these social processes. Network parameters are useful to compare the network structure across different time points [19] or to measure the impact of an intervention [20]. The maps of relationships (sociograms) are highly engaging to the participants and can be used as an intervention to encourage strategic linkages [21].

Interconnections between agents are a key feature of complex systems, which social network research allows us to examine in greater detail [22]. We used an online, whole network study to examine this aspect of complexity among members of Australian Genomics, asking respondents about two timepoints: pre-existing relationships (pre-2016) before the formation of Australian Genomics and current (2018), after 2 years of operation. By measuring the pre-existing collaborative ties that were formed outside of Australian Genomics and then those that were formed as a result of Australian Genomics, we can assess the extent of change, exemplified by new tie formation. If the collaboration is successful, new ties may be expected across clinical specialties, genetic and non-genetic specialties, and State and Territory boundaries.

### Aims

The primary aim of this study was to map and analyse the collaborative interconnections of members of Australian Genomics, to capture the intuitive but elusive concept of the emerging socio-professional genomic community, capable of collective learning: a knowledge exchange network. A second aim was to assess the influence of Australian Genomics’ strategy in bringing players from different fields together. Third, we sought to identify key players in the Australian Genomics network (central actors and brokers), isolated members or groups, and the reach of this genomic community (i.e., outside Australian Genomics) within Australia and overseas. This analysis will assist in identifying risks and opportunities for the network moving into the future, with the ultimate goal of getting genomics into routine practice.

## Methods

### Design

This was an online, whole network study. Whole network studies rely on high response rates, especially from people known to be highly involved in the network. The data collection tool was therefore named the *Australian Genomics Census* to indicate that we were trying to capture the entire membership, not just a sample. It was framed as a key Australian Genomics activity for its members.

The *Australian Genomics Census* was designed by an expert panel made up of health services researchers with network expertise and key informants from Australian Genomics. Since social network studies seek to map an appropriate relationship of interest, the panel discussed the wording and framing of the tie question at length. Suggestions included who do you “work with”, “seek information or advice from” or “collaborate with,” all in the context of genomic clinical practice. The observation from the panel that members were learning how to practice genomics from one another through working in new informal teams suggested the idea of a learning community. The key relationship of interest was therefore framed around “who is part of your genomic learning community / knowledge exchange network?” The expert panel also defined the boundaries of the network, i.e., the final list of invited participants who were considered members of Australian Genomics. Members were defined as people participating in a Flagship Project, Program working group, Governance body (e.g., National Steering Committee, Community Advisory Group), or being Operational staff (e.g., Project Officers, Manager). Information on each member was collated to assist analysis: State or Territory of residence, profession and primary group membership. Primary role was used for members holding hybrid professions (e.g., clinicians who also undertook research). The *Census* was designed on the survey platform Qualtrics, securely hosted on the administering University’s computer server.

### Participants, recruitment and confidentiality

Invitations to participate in the *Census* were sent on behalf of the researchers by Australian Genomics during May 2018 after it had been publicised in meetings and official communications for the three preceding weeks. Australian Genomics members were individually emailed a unique link to the census and were encouraged to participate in the 10 min structured questionnaire. Information about the project and its purpose were provided in the invitation and the first question of the *Census* asked for formal acknowledgement of consent. Information given in the email explained that social network questionnaires map the relationships between participants by surveying as many members of the network as possible and then combining their answers. In order to construct the network, it is necessary to use people’s names so the links can be matched. Once submitted, data was coded (e.g., participant #22, #198) and names were removed. Our aim was to ensure individuals, programs and institutions would be anonymous in all wider reporting of results and would use aggregation of small groups to assist anonymity. Leaders of groups were asked to encourage completion by their members, and up to two reminders were sent out to non-responders at 2-week intervals.

### Measures

#### Relational data

Participants were asked to provide data on working relationships within their team and between their team and other Australian Genomics members. The social network question was phrased:

Please work your way down the list [of names] and indicate who you work with on Australian Genomics projects. By “work with” we mean in the context of genomics **–** shared care of patients, worked in the same lab, been involved in research together, participated in a working group together, had a phone call about Australian Genomics etc. We do not mean people whom you know only by reputation (e.g., heard them speak at a conference, read a journal article authored by them). **We are trying to capture the idea of a socio-professional genomic community, capable of collective learning: a knowledge exchange network.**

As the number of members in Australian Genomics was so large, we structured the roster of names by Program working group, Flagship project, or other group (e.g. the National Steering Committee, Community Advisory Group). Participants were asked to select the group, or groups in which they took part. This gave them access to the list of people involved in that group who we expected would be their most likely collaborators. There was no limit to the number of groups that could be selected. The final choice in this section was an alphabetical list of everyone in Australian Genomics for people who were not sure which group a working colleague was listed. Students were not listed by name in the roster of Australian Genomic members as a complete list of students was not at that time available. Participants who were working with students on Australian Genomics projects were asked to name them in a free text box.

For each person nominated as someone with whom the respondent worked on Australian Genomics projects, respondents were asked “Did you know this person before you joined Australian Genomics?” Respondents could select either “I knew this person before I joined Australian Genomics” or “I only met this person through Australian Genomics”.

Two additional questions asked participants to list external collaborators from outside Australian Genomics, but within Australia, whom they considered part of their socio-professional genomic community. There was room for up to ten names. A second question then asked for external collaborators from outside Australia.

#### Sources of information

Three questions asked participants to rate the extent that differing sources of information had informed their genomic work, specifically towards getting genomics into routine care. Formal sources of information (including journal articles, formal courses or short presentations at work), informal information (e.g., learning by doing, trial and error) and group influences (e.g., governing body, consumer advisory group). Each option was rated using a five-point Likert scale from “not at all” to “to a large extent.” A “not applicable” option was also included.

### Analysis

Social network data was analysed and visually constructed using Gephi 0.9.2. Network parameters of density (the proportion of actual ties to possible ties), centrality (a measure of who is the most interactive member) and brokerage (a measure of who are the key people acting as go-betweens by virtue of their position linking people together) were computed. Differences between the pre-existing network (pre- 2016) and the current new network catalysed by Australian Genomics (2018) were analysed. Likert scale responses were analysed in SPSS v22 using descriptive statistics and *χ*^2^ analysis.

### Ethics

Ethical (institutional review board) approval for the project was granted by the administering University’s Human Research Ethics Committee (5201701186). The *Census* was authorised by the Australian Genomic Health Alliance National Steering Committee.

## Results

### Participants

Invitations were emailed to 384 members of Australian Genomics. The response rate was 57.81% (222/384). Three respondents formally declined to do the *Census*. A comparison of respondents and non-respondents revealed no significant differences in the gender distribution of the network: *χ*^2^(1, *N* = 384) = 1.17, *p* = .28. However, there was a significant difference in the representation of professional groups when comparing respondents and non-respondents; *χ*^2^(1, *N* = 387) = 31.56, *p* < .05, specifically for Medical Specialists and Genetic Specialists (see Table 2). Representation from the different States and Territories reflected overall membership: *χ*^2^(1, *N* = 384) = 8.33, *p* = .40.

**Table 2:** Comparison of respondents and non-respondents of the *Australian Genomics Census* showing chi-squared analysis

	Total (*N*)	Respondents (*n*, %)	Non-respondents (*n*, %)	*χ* ^2^
Females	202	122, (60.39%)	80, (39.60%)	*χ*^2^(1, *N* = 384) = 1.17, *p* = .28
Medical specialists	73	25, (34.25%)	48, (65.75%)	*χ*^2^(1, *N* = 384) = 20.52, *p* = < .05*
Genetic specialists	94	71, (75.79%)	23, (24.21%)	*χ*^2^(1, *N* = 384) = 16.02, *p* = < .05*
Medical scientists	100	52, (52.94%)	48, (44.12%)	*χ*^2^(1, *N* = 384) = 1.87, *p* = .17
Researcher^	42	27, (64.29%)	15, (35.71%)	*χ*^2^(1, *N* = 384) = 0.81, *p* = .37
Other	75	47, (62.67%)	28, (37.33%)	*χ*^2^(1, *N* = 384) = 0.90, *p* = .34

*p < .05 significant. ^“Researchers” include biomedical, health services and sociological researchers, and “Other” includes operational staff, students and consumers

### Relational data

#### Australian Genomic members

Four sociograms were constructed to map the collaborative working relationships of Australian Genomics members, considered to be their genomic learning community: (a) all links across members, (b) collaborative links with people known to the respondents before Australian Genomics started operation, (c) new collaborative links with people not known to the respondents before Australian Genomics started operation and (d) collaborative links to genomic partners external to Australian Genomics but within Australia. Table 3 summarises the computed parameters for each of the four networks. Collaborative links to genomic partners external to Australian Genomics from outside of Australia were collated.

**Table 3:** Computed parameters for four networks: Australian Genomics learning community 2018, “pre-existing ties pre-2016”, “met through Australian Genomics” and external collaborators from within Australia

Parameter	Australian Genomics learning community 2018	“Knew before” network (pre-2016)	“Met through” network	External collaborators in Australia
Number of nodes	384	384	384	412
Number of informants*	209	203	174	93
Number of ties	6381	2925	3351	464
Number of isolates	5	27	20	NA**
Highest in-degree	91	44	75	7
Highest out-degree	354	87	338	10***
Centralisation	0.886	0.208	0.864	NA**
Density	0.043	0.020	0.023	0.003

*Number of census respondents providing the information. **Not applicable as only included respondents who answered this question. ***Name generator capped at 10

**Fig. 1:**
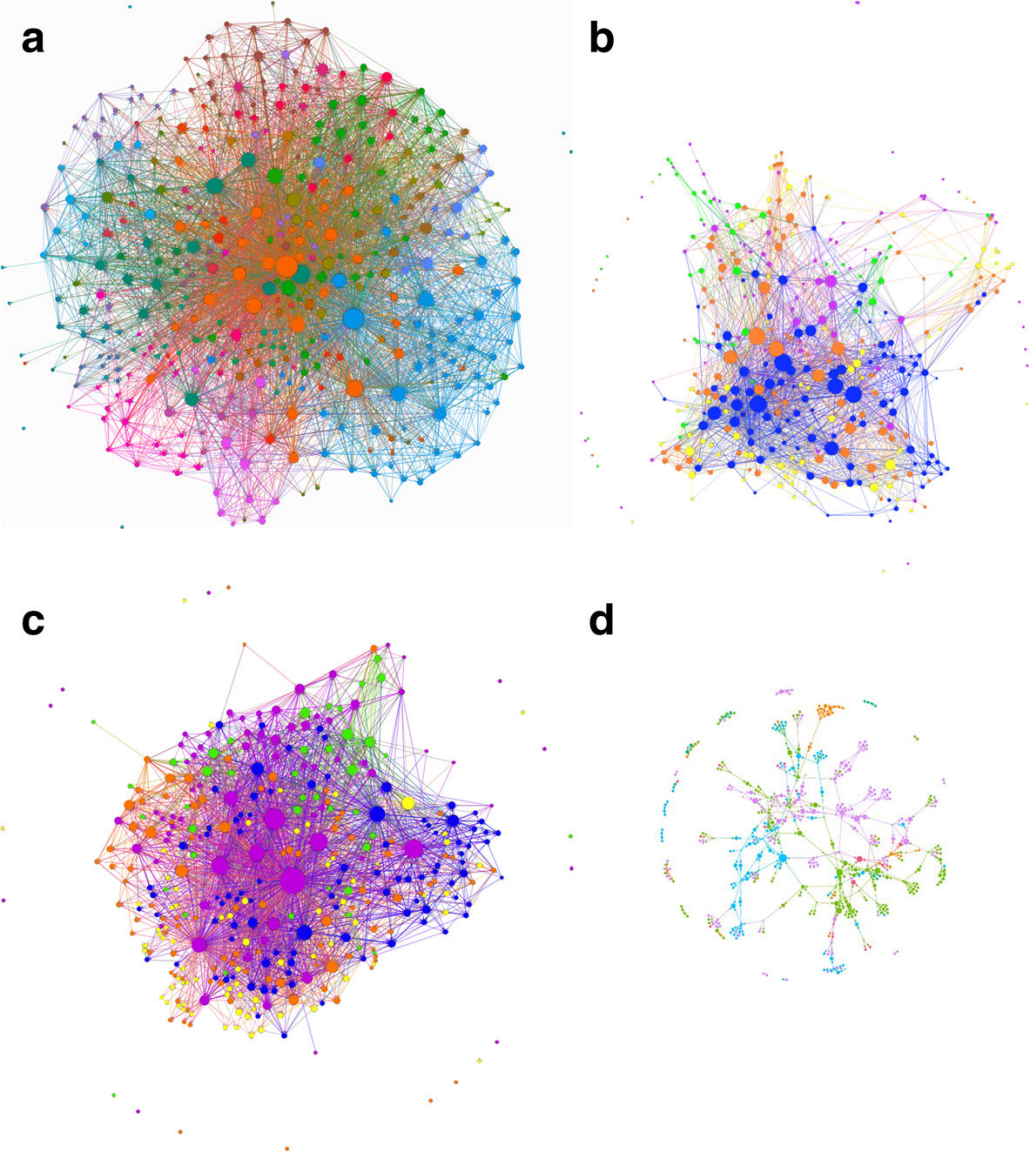
Sociograms. Nodes are Australian Genomic members and size of node is indicative of in-degree (the bigger the node the more highly nominated). **a** Australian Genomics socio-professional network. Colours show the respondents’ groups (seven groups with the most ties of a total of 38 group). Legend:  Operations,  KidGen Renal Genetics,  Acute Care Genomic Testing,  Genetic Immunology,  Cardiovascular Genetic Disorders,  National Steering Committee,  Acute Lymphoblastic Leukaemia. **b** Genomic learning community of respondents before Australian Genomics Health Alliance started operation in 2016. Colour of node shows professional qualification of the respondent:  Medical scientist,  Genetic specialist,  Other (e.g. consumer, student, operational staff),  Medical specialist,  Researcher. **c** “Met through Australian Genomics” network: genomic learning community in 2018 after 2 years’ operation of Australian Genomics Health Alliance. Colour of node (see **b**) shows professional qualification of respondent. **d** Collaborators from outside Australian Genomics, resident in Australia. Nodes here are respondents to this question and the people they nominated. Colour of node indicated State or Territory of Australia:  New South Wales,  Queensland,  Victoria,  South Australia,  Australian Capital Territory,  Western Australia, Tasmania

Figure 1a shows the sociogram of collaboration and learning between all Australian Genomics members. The network was constructed from the responses of 209 participants who nominated 379 members. Respondents reported 6381 ties to other members whom they considered part of their genomic learning community. The density (the proportion of actual ties to possible ties) of this network was 0.043, and there were five isolates (i.e. people who did not nominate anyone nor were nominated themselves by anyone). The network contained a single component (excluding the isolates) showing that there are no completely isolated state-based or profession-based clusters but links between them all. External-Internal index (E-I), a number between − 1 and 1, measures the probability that ties are formed at random across groups. This was computed with State or Territory of residence as the groups and was found to be significant (*p* < 0.05) at − 0.385. This indicates that ties were more likely with members from their own State or Territory than outside it.

The second sociogram (see Fig. 1b) shows only the links of members who had known each other prior to 2016, before Australian Genomics started operation. There were 203 respondents who reported 2925 collaborative ties, the network consisted of 27 isolates, and the density of ties was 0.020.

The third sociogram (see Fig. 1c) shows only new collaborative links: ties of members who had only met each other through Australian Genomics over the past 2 years. There were 174 respondents who reported 3351 collaborative ties. There were 20 isolates in this network and the density of ties was 0.023.

#### External collaborators

The sociogram of external collaborative partners within Australia was constructed from the responses of 93 participants who nominated 328 people in seven States or Territories (no collaborators were nominated from Northern Territory) of Australia. Figure 1d shows the sociogram of external collaborators within Australia.

**Fig. 2:**
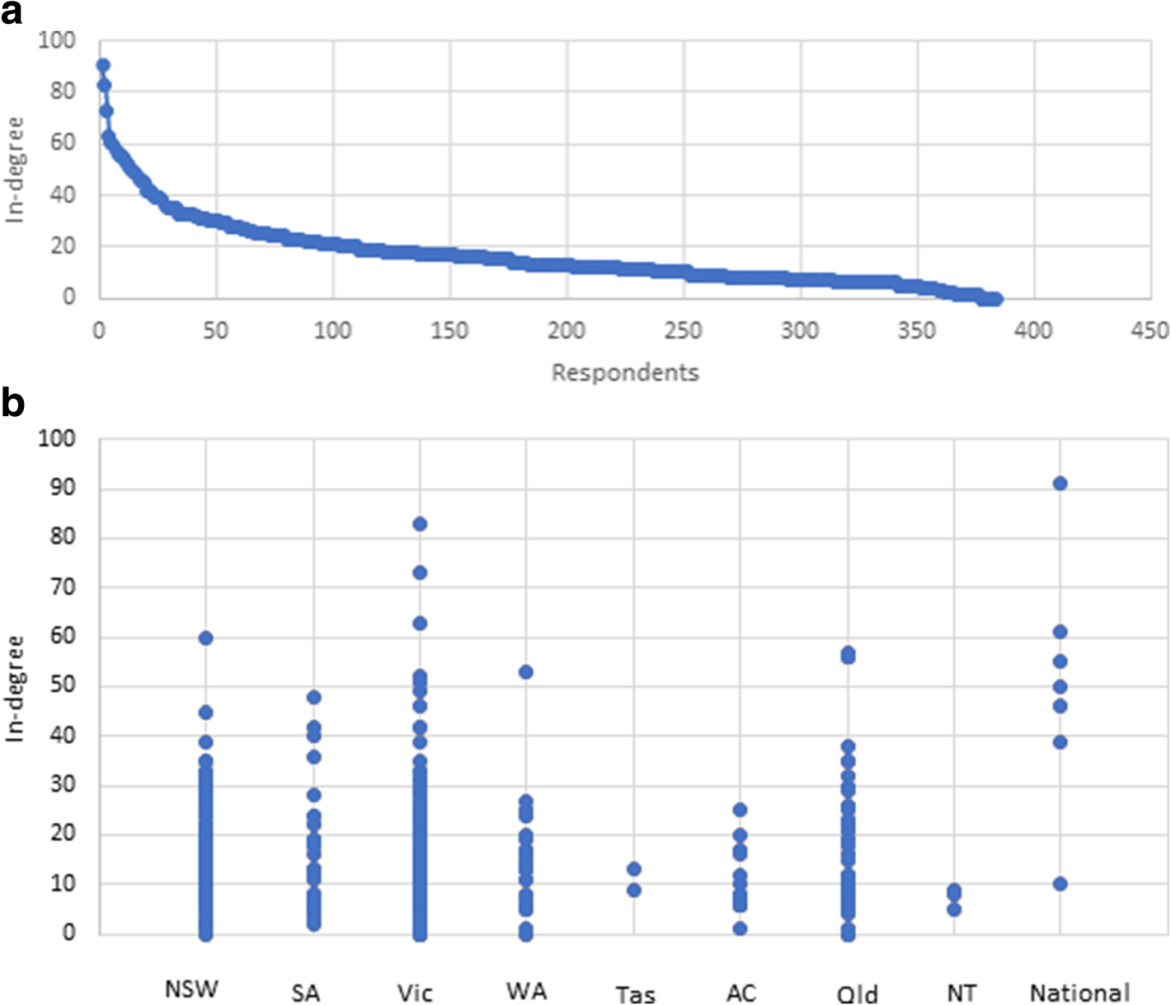
**a** Distribution of in-degree for the Australian Genomic community. In-degree counts the number of ties directed to an actor in the network and is a measure here of influence. **b** Dot plot showing the distribution of in-degree by State of Australia. (NSW, New South Wales; SA, South Australia; Vic, Victoria; WA, Western Australia; Tas, Tasmania; ACT, Australian Capital Territory; Qld, Queensland; NT, Northern Territory)

Respondents nominated 355 people from 24 different countries as external collaborative partners from outside of Australia. Collaborators were most frequently from the USA (*n* = 113) and the UK (*n* = 100). Six countries had only one collaborator each.

### Key players

Key players within the Australian Genomics community were identified as those members having the highest ranked in-degree (number respondents who have nominated the focal member), out-degree (number of members nominated by the focal member) or betweenness centrality (a measure of how influential the focal member is as a broker or go-between). Centralisation, a network level measure of how focussed on key players the whole network is, was also computed. In any of the degree measures examined, ranges of values had a marked positive skew and between one to five outliers (see Fig. 2 for an example). Outlying actors were considered the top-ranking players (i.e. most interactive members) in the Australian Genomic network. Table 4 summarises the key players.

**Table 4:** Key players in the Australian Genomics socio-professional genomic community identified as outliers (see Fig. 2a)

Network parameter/attribute	Measure	Profession/role	State
In-degree/most influential	91	Australian Genomics Manager	National
	82	Clinical geneticist	Victoria
	73	Clinical geneticist	Victoria
Out-degree/most connected	354	Australian Genomics Manager	National
	260	Operational staff	National
	210	Project Officer	South Australia
	207	Clinical geneticist	Victoria
	162	Project Officer	National
Betweenness centrality/brokers	13.39	Australian Genomics Manager	National
	5.59	Clinical geneticist	Victoria
	3.02	Operational staff	National

#### Sources of information

**Fig. 3:**
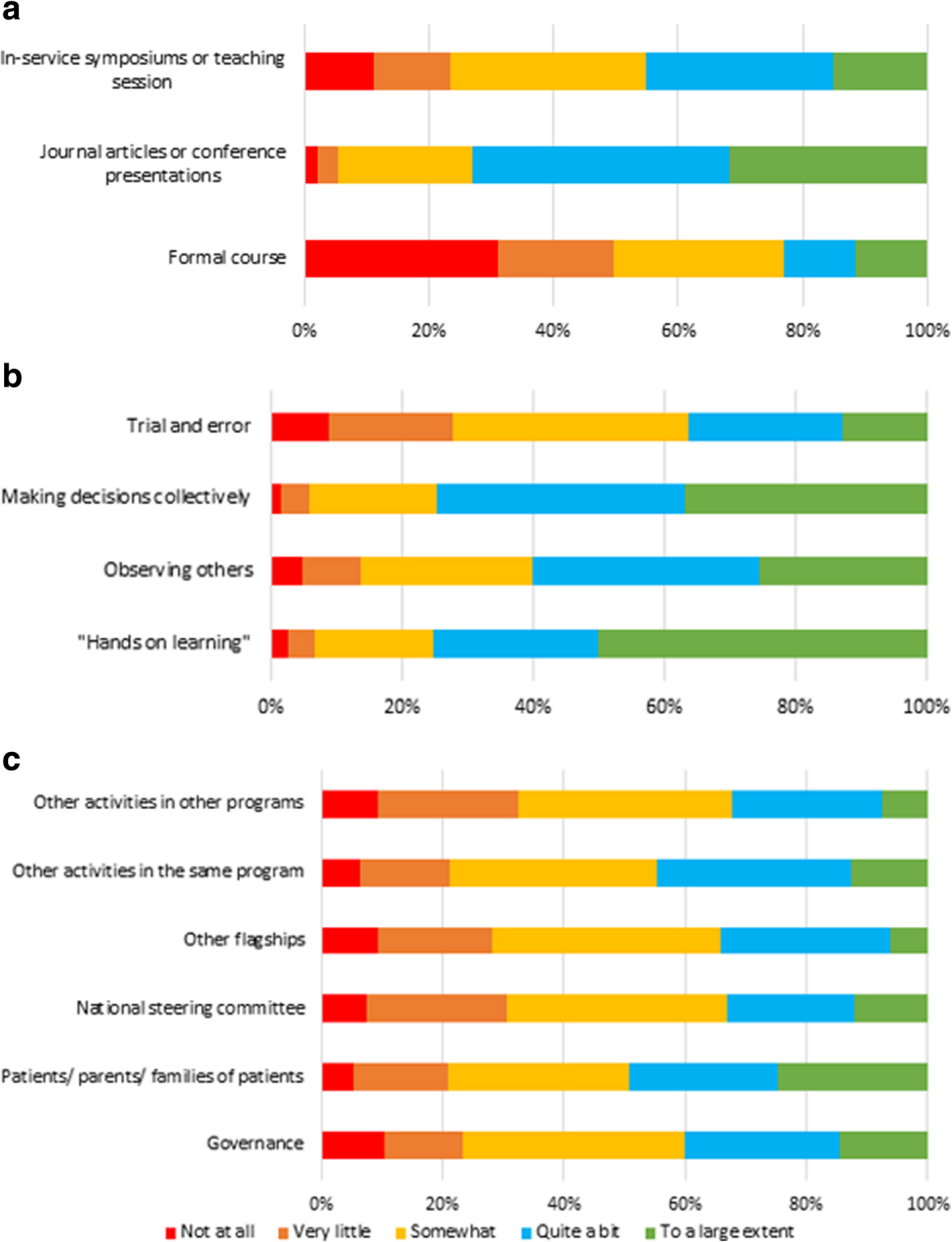
Extent to which different factors Influenced respondents’ genomic practice. **a** Formal sources of learning. **b** Informal sources of learning. **c** Australian Genomic Groups and stakeholders groups

Among the categories of information sources, *formal courses* were the least likely to be sources of genomic information for respondents (50% “Not at all” or “Very little”). Use of *journal articles or conference presentations* was the most common formal source of genomic information (73% “To a large extent” and “Quite a bit”). Figure 3a shows this graphically.

Among informal sources of information, *hands on learning* was by far the most popular response (50% “To a large extent” and 25% “Quite a bit”), followed by *making decisions collectively* (37% “To a large extent” and 39% “Quite a bit”). *Using trial and error* had the highest frequency of “Not at all” (9%) or “Very little” (19%). Figure 3b shows this graphically.

When participants were asked about group influence on their genomic practice, the most popular response in every category was “Somewhat” (30–36%). Figure 3c shows this graphically.

When comparing the extent to which differing sources of information had informed participants’ genomic practice between members in Flagship projects (e.g. Kidgen renal genomics, Mitochondrial diseases) compared to members in other groups (e.g. National Steering Committee, Health Economics), no significant differences were revealed (*p* > .05).

## Discussion

This study used social network methodology to uncover systems complexity by describing and analysing the genomic learning community of Australian Genomics members. Australian Genomics appeared to be driving a significant number of new collaborative relationships in the 2 years since it started operation. Key players were operational staff or clinical geneticists. The finding that social processes, notably hands on learning and group decision-making, are perceived as the greatest influences on members’ genomic practice, underlines the importance of Australian Genomics’ work in facilitating the emergence of a genomic learning community.

### Australian Genomic learning community

The interactions of members of Australian Genomics and the genomic learning community were the focus of our study. While our research revealed the shifting nature of relationships in the changing CAS, quantitative social network analysis can only describe the presence or absence of ties not the motivations behind their formation. However new ties are likely formed to access needed expertise. Generally speaking, single practitioners cannot use clinical genomics; it is a highly interdisciplinary pursuit. This is likely to be one of the strongest drivers for participation over time.

People from traditionally insular disciplines are now cooperating and learning from one another: medical specialists were being brought together as genomic testing cuts across silos based on organ group, medical condition or stage of life, and different professions are collaborating across the normal boundaries of research laboratories, testing laboratories, genetic departments and clinical groups. With the development of this national initiative, boundary-spanning roles have emerged, such as bioinformaticians and implementation scientists, each with an area of expertise to contribute to the effective use of clinical genomics: selecting appropriate patients to test; providing an informed consent process, accurate analysis, robust interpretation of variants and the clinical management implications; and returning the results to the patients and their families. A Community Advisory Group and National Advisory Group convened by Australian Genomics also bring valuable insights from the perspective of patients, carers and community members.

### Measures

This study did not control for external interventions that occurred during the same time period that may also have been driving collaborative ties, so causality cannot be definitively established. However, evidence that Australian Genomics is perturbing the extant system, and driving this learning community, is the change in the number of ties seen between the “knew before” network (i.e. pre-2016) and the current 2018 “met through Australian Genomics” network. There is a notable increase in the density and the number of ties. Moreover, the “knew before” network has low network centralisation most likely reflecting the national context of State-based Genomic Alliances. Not surprisingly, the “met through Australian Genomics” network is much more centralised, showing the strong influence of the operational staff and the network manager in linking up clusters from across the States and Territories. Geography is still a dominating factor in this network. Meaningful collaboration is driven by proximity [23], and teams delivering patient care might be expected to work closely together. Funding linked to state-based genomic projects will also be a factor in determining collaborations.

Density of the current 2018 network (0.043) is higher than the 2016 (“knew before”) ties network (0.020) indicating an increase in connectivity among these members. Density measures in a social network such as this reflect the necessary trade-off between the benefits and costs of collaboration. The benefits are new ideas and knowledge, but the cost is time to maintain that relationship [24]. Maintaining too many ties may become onerous and counterproductive. There is known value in boundary-spanning ties as a source of innovative ideas from beyond the boundary of one’s close network [25]. The mixing of professions seen here shows this is happening across professions and to a lesser extent across States but the strength of a cohesive team is perhaps more conducive to collective learning [26].

Australian Genomics’ facilitation of socio-professional linkages, while substantial, is not the only influence on clinical genomics. Other significant drivers outside Australian Genomics are the ever-growing number of research outputs both in Australia and overseas requiring cooperation between contributors and rapidly advancing genomic technology [27]. State-based Genomic Alliances also are running successful programs and facilitating knowledge exchange within their States.

Within Australian Genomics, Program work, infrastructure and funding support are likely to be significant drivers of increased collaborative ties. The availability of competitively awarded funding for Flagship projects has spurred some individuals to seek out and assemble expert teams with coherent project plans in order to access funding.

### Key players

Key player analysis showed the central role of the Australian Genomics Manager and two clinical geneticists. These key players had the highest in-degree, meaning they were nominated the most as part of respondents’ genomic learning community. Clinical geneticists are active members in many of the working parties and especially the Flagship Projects, valued for their specialist knowledge of genetics, so their centrality in a learning community is not unexpected.

The Australian Genomics Manager had the highest in-degree, out-degree and brokerage score. As found in other similar networks [19, 28], the manager’s role in a number of different working groups and Flagship projects makes them highly visible and knowledgeable about the whole alliance.

The manager is also the obvious “go-to” person for information concerning funding opportunities. Moreover, the high profile of the Manager in attending Program and Flagship meetings means she is accessible to many members. The central coordinating and brokerage role that this manager is enacting contributes significantly to the success of the network functionality.

The effectiveness of central actors (here, the network manager, operational staff and clinical geneticists) as knowledge brokers is usually a combination of both their expertise and their accessibility [29]. We did not explore here whether these central actors are nominated because they are available, or because they have the sought after expertise, or because they are likely to be able to direct enquiries to the appropriate person (acting as a broker). As seen in other networks [19], there are risks here for a key player to be overloaded with requests, or to leave the network, leading to the potential for network fragmentation or for a regression of actor behaviour back into silos, or for a slowing down of collaborative working [19, 28]. Mitigation strategies include sharing the particular role they are enacting (brokerage or source of expertise) across other members, or diffusing responsibilities across the network. For routine requests that the network manager might field, making information available via electronic means may also be considered*.*

Operational staff (Australian Genomics employed Project Officers and Managers) also figure highly on the key player list, notably as people with high out-degree, a measure of their connectedness to others. Hiring operational staff to communicate across network activities and encourage collaboration was one of Australian Genomics’ tactics to facilitate Flagships and Program activities. From inception, they were empowered to be “ambassadors” for Australian Genomics. Operational staff are known to be influential connectors within similar networks [19, 30]. Their role affords them a broad overview of members and members’ expertise and gives them awareness of specific needs individuals may have (i.e., for resources or research partners). This allows them to act as intermediaries and introduce the two parties. Personal introductions such as these are valued highly and are more likely to result in a fruitful collaborations than a more objective process, say, self-selection of a partner from a website list [31].

Figure 2b shows that Victoria, New South Wales, Western Australia, Queensland and South Australia each have respondents who are shown as outliers who are significantly more influential than the rest of the respondents in their State. This suggests pockets of strong influence spread across these States.

### External collaboration

Australia is, of course, not the first country to introduce genomic medicine into health services. There are many national implementation consortiums such as Genomics England [32] and international alliances such as the Global Genomic Medicine Collaborative [33] which have been in operation longer than Australian Genomics. The imperative for global data sharing of genomic data, especially for genes implicated in rare diseases, is considered essential to meet the aims of individual countries [34]. It seemed likely that Australian researchers would be learning from, be influenced by, and be collaborating with key players from overseas and this was borne out in our results. The 93 respondents to this question nominated 355 people from outside of Australia, and this is likely to be an underestimate of true connection levels. Respondents could name up to 10 overseas collaborators. Since 17 respondents filled all 10 spaces, it is possible that key people may have been able to nominate many more than 10.

The largest number of overseas collaborators is from the USA (*n* = 113) followed by the UK (*n* = 100). Collaborative links from the UK reflects not only a common language and heritage but a similar health system based on a universal insurance model. The predominantly private health system of the USA in which insurers are key stakeholders makes many aspects of implementation different, but a large population and generous funding for genomic research also makes individuals working in this system valuable collaborative partners.

Many people named as collaborators within Australia but external to Australian Genomics were inside Australian Genomics according to our definition. This implies that Australian Genomics has unclear borders for some. Possibly, some of the people nominated have joint affiliations with a state-based genomic alliance or had only recently moved to a new position within Australian Genomics.

#### Influences on genomic practice

It is clear from the questions around influences on the respondents’ genomic practice that self-directed activities and social and professional processes predominate. Among the formal sources of influence, self-directed learning from journal articles or conferences and in-service education were the more popular sources. Formal courses were rated “Not at all [influential on my genomic practice]” by 31% of respondents. A formal course was not defined in the question, so we do not know if respondents were thinking of their graduate degrees or specialist training and stating that they had not been helpful, or that more recent, genomic-focussed courses had not been useful.

Among the more informal sources of influence, hands on learning was clearly the most popular with 50% of respondents reporting it influenced their genomic practice “To a large extent,” followed by “Making decisions collectively” with 46%, both socially based processes. In contrast, the social process of being influenced by groups outside one’s immediate team seems less potent. The response to most of the groups listed was lukewarm at best. We tested whether clinically based respondents (which we identified as people working in a Flagship genomics program) might have answered differently to those outside (who we identified as people in non-clinically based working groups or committees), but no significant correlation was found. This could suggest that people with direct patient contact were influenced by much the same factors as people in other positions, such as Community Advisory Group members or laboratory scientists. On the other hand, many of the members of the various governance committees have dual or triple roles: scientists or clinicians also involved in Flagships or Programs and this may suggest that clinical experience was being shared across all groups.

#### Recommendations

The observation from our study that respondents consider learning in the genomic context to be predominantly a self-directed, social process, combined with the evidence of tie formation across medical specialty and disciplinary silos, has implications for genomic networks, implementation efforts and education strategies.

- Genomic networks should recognise the value of and actively foster the formation of interdisciplinary learning communities carrying out genomic medicine alongside their support of smaller interdisciplinary teams. Many papers have emphasised that clinical genomics requires the formation of new, small, interdisciplinary teams that combine medical scientists, genetic and medical specialists (e.g., [35]). Our study showed that participants included in their genomic learning community members from other Flagships (meaning members working within another specialty/body system or life-stage) and Programs (including, e.g., implementation scientists and ethicists) showing that links outside the traditional silos of medical specialty and field are also considered important.
- Educational resources should be tailored to support self-directed and group learning, favouring interactive material over passive information giving sessions. Paul and colleagues [36] note in their systematic review of factors affecting clinicians’ genetic testing practices that clinician education was the only strategy proposed in 24 of the 39 studies identified, and further note that there was scant evidence of its success. Our findings confirm that formal courses are ranked as weak influencers of practice. Members reported self-directed, experiential learning as more effective implying a slower process of up-skilling that needs appropriate resourcing.
- Service providers and managers should allow quarantined time for both up-skilling and implementation of genomic practice. Building of genomic knowledge and skills through hands on experience and mentoring across traditional silos will take time and needs to be seen as additional to routine service delivery. Moreover, the perceived importance of group decision-making and experiential learning reflects the evolving, emergent nature of the genomic practice field. There are not yet clear protocols to direct practice and the wealth of new research on clinically relevant gene variants presents significant challenges. The importance of varied expertise within teams to provide specialist knowledge and the usefulness of supportive technologies in uncertain situations should be recognised and supported.

#### Limitations and strengths

Two limitations of social network designs are their reliance on self-report and issues of missing data. Our use of a name interpreter design, that is, providing a list of Australian Genomic members, assisted recall and enabled respondents to quickly select ties. However, the pragmatic decision to format the names of members by working groups or Flagships may have introduced artificial clustering, especially if respondents did not go on to select other groups with whom they were working.

Care was taken to ensure the relationship question was not ambiguous in any way: crafted over several sessions by an expert panel and incorporating feedback from piloted versions. Missing data from non-respondents is partly mitigated by the answers of other respondents. If Person X does not take part but Person Y does and nominates Person X as a collaborator, Person X will be included. In this way, all 384 members could be represented in the sociograms. Whole network studies are most affected by missing data when key players do not respond. If we consider operational staff (*n* = 5), and project leads (*n* = 31) as being the most active in Australian Genomics, all but three of these people responded, giving us confidence the data is adequate, with sufficient fidelity to be meaningful. This was partly achieved through a targeted reminder email sent from Australian Genomics.

## Conclusion

This study applied social network methodology to uncover and document the dimensions of Australia’s genomics community, an emergent CAS with properties that mirror other complex systems. Australian Genomics was shown to be a catalyst for collaborative tie formation. The Australian Genomics’ Manager was identified as the key player facilitating collaboration and sharing of expertise across the national network. Other key players were operational staff and two clinical geneticists, people who have roles across a number of different working groups or projects. Social processes and self-directed modes of learning were shown to be powerful influences on members’ genomic practice, underlining the significance of the strategy of building relationships to form a genomic learning community. These findings have implications for implementation, education and workforce strategies going into the future.

## Abbreviations

CAS: Complex adaptive system
E-I: External-Internal index
